# Improving access to care in low and middle-income countries: institutional factors related to enrollment and patient outcome in a cancer drug access program

**DOI:** 10.1186/1472-6963-13-304

**Published:** 2013-08-10

**Authors:** Ebru Tekinturhan, Etienne Audureau, Marie-Pierre Tavolacci, Patricia Garcia-Gonzalez, Joël Ladner, Joseph Saba

**Affiliations:** 1 Axios International, 7, Boulevard de la Madeleine, Paris 75001, France; 2 Biostatistics and Epidemiology Unit, Hôtel Dieu, Assistance Publique Hôpitaux de Paris, Université Paris Descartes, Sorbonne Paris Cité, Paris, France; 3 Department of Epidemiology and Public Health, Rouen University Hospital, Rouen, France; 4 The Max Foundation, Edmonds, WA, USA

**Keywords:** Cancer, Access to medication, Enrollment, Implementation, Survival

## Abstract

**Background:**

Limited access to drugs is a crucial barrier to reducing the growing impact of cancer in low- and middle-income countries. Approaches based on drug donations or adaptive pricing strategies yield promising but varying results across countries or programs, The Glivec International Patient Assistance Program (GIPAP) is a program designed to provide imatinib free of charge to patients with chronic myeloid leukemia (CML) or gastrointestinal stromal tumors (GIST). The objective of this work was to identify institutional factors associated with enrollment and patient survival in GIPAP.

**Methods:**

We analyzed follow-up data from 4,946 patients participating in 47 institutions within 44 countries between 2003 and 2010. Active status in the program was considered as a proxy for survival.

**Results:**

Presence of ≥1 hematologist or oncologist at the institution was associated with increased patient enrollment. After adjusting for individual factors such as age (>55 years: Hazard Ratio [HR] = 1.42 [1.16; 1.73]; p = 0.001) and initial stage of disease (accelerated or blast crisis at diagnosis: HR = 4.16 [1.87; 9.25]; p < 10^-4^), increased survival was found in institutions with research capabilities (HR = 0.55 [0.35; 0.86]; p = 0.01) and those with enrollment of >5 patients/year into GIPAP (HR = 0.48 [0.35; 0.67]; p < 10^-4^), while a non-significant trend for decreased survival was found for treatment at a public institution (HR = 1.32 [0.95; 1.84]; p = 0.10). The negative impact of an accelerated form of CML was attenuated by the presence of ≥1 hematologist or oncologist at the institution (interaction term HR = 0.43 [0.18; 0.99]; p = 0.05).

**Conclusions:**

Application of these findings to the support and selection of institutions participating in GIPAP may help to optimize care and outcomes for CML and GIST patients in the developing world. These results may also be applicable to the treatment of patients with other forms of cancer, due to the overlap of infrastructure and staff resources used to treat a variety of cancer indications. A multi-sector approach is required to address these barriers.

## Background

The incidence of cancer is increasing globally, particularly in low-and middle-income countries [1-5]. The increased incidence of cancer in these countries is essentially due to a combination of a decrease in deaths from infectious diseases and an increase in average population age [1,3,4]. It is estimated that the proportion of cancers diagnosed in low- and middle-income countries will rise over the next two decades, ultimately accounting for approximately 70% of cases globally [1,4]. There are a number of challenges to reducing the impact of cancer in low- and middle-income countries, including a lack of effective cancer surveillance and control, lack of trained cancer specialists, lack of diagnostic and treatment capacity, few functional cancer registries, scarce and expensive cancer medicines, as well as a general lack of adequate health care, effective disease prevention policies and health care funding [4,6]. The high cost of newer, more targeted therapies for cancer also creates a significant barrier to providing cancer patients in low- and middle-income countries with the latest advances in care treatment [1,5].

The growing impact of cancer in low- and middle-income countries calls for new approaches to preventing and treating this disease [1]. While limited data are available for access programs that target non-communicable diseases, the success of various efforts to increase access to HIV therapies provides several models that may be applied to cancer and other chronic diseases [1,5,7,8]. Such efforts include integration of HIV care with other health and social services, reduced and not-for-profit pricing of HIV medications, drug donations and a variety of partnerships among national governments, non-governmental organizations (NGOs) and pharmaceutical industries [5,9,10]. Several innovative strategies for reducing the impact of cancer in low- and middle-income countries have already been implemented and are showing promise in improving access to care and patient outcomes [6,11-15]. These efforts include: decreasing cancer risk factors (such as tobacco exposure), development of guidelines for establishing national cancer programs, leveraging existing cancer research capabilities to establish additional cancer-related programs, establishment of national or regional referral centers, use of telemedicine to improve access in remote areas, increasing access to cancer screening, drug donations and adaptive pricing strategies in which patients pay what they can afford and have the rest of their medication costs covered by other organizations [6,11-13,16].

Glivec (imatinib), a small molecule inhibitor of the bcr-abl tyrosine kinase that results from the Philadelphia chromosome (Ph+), has been shown to improve progression-free survival in patients with Ph + chronic myeloid leukemia (CML) [17]. The annual incidence of CML is estimated at 0.6-2.0 people per 100,000, and the prevalence of CML appears to be increasing, potentially due to increased survival resulting from treatment with imatinib [11,18,19]. Clinical studies also support the utility of imatinib in the treatment of gastrointestinal stromal tumor (GIST) [17]. While imatinib has been shown to improve outcomes for CML and GIST patients, its average cost of $2,500 to $3,500 USD per month could limit its use in low- and middle-income countries.

The Glivec International Patient Assistance Program (GIPAP) began in 2001 through the support of Novartis, the manufacturer of imatinib, in conjunction with Axios International, The Max Foundation and several other NGOs. The program is designed to expand access to imatinib among patients with CML or GIST. The program has been implemented in 81 developing countries-including 49 Least Developed Countries (LDCs) as defined by the United Nations–and reached 50,395 patients who have received Glivec through GIPAP free of charge.

A previous study has shown that patients participating in GIPAP are more likely to improve their phase state, regardless of disease stage at the time of entry into the program, and that patients in the program show high survival rates after 2 years, comparable with data from a randomized phase III trial [11,20,21]. The authors of this study identified several differences between countries with respect to the magnitude of the improvements in the health state achieved by patients enrolled in GIPAP, although these trends did not reach statistical significance. This suggests that important factors nested at the national or institutional level could influence outcomes at the patient level. In this respect, both the overall organization of a country’s national health system (i.e. health insurance coverage, access to healthcare) and local characteristics, such as research ability or public/private status of the institutions and hospitals at which GIPAP has been implemented could play a significant role in patient outcomes. It is still not known if the capacity of a given institution to enroll patients into GIPAP efficiently and on a regular basis could be associated with better outcomes for patients in the program.

In order to address these important questions, the objectives of this study were to 1) describe the patients and the institutions that participated in GIPAP, 2) assess enrollment rates at the institutional level and identify their possible determinants and 3) identify patient and institutional factors associated with patient survival.

## Methods

The requirements for participation in GIPAP have been described previously [11]. Briefly, patient eligibility is determined primarily on the basis of diagnosis, as well as income/socioeconomic status, as follows: (a) GIPAP helps patients who are properly diagnosed with Philadelphia chromosome-positive chronic myeloid leukemia (Ph + CML) and patients with c-Kit (CD117)-positive inoperable and/or metastatic malignant gastrointestinal stromal tumors (GISTs) and (b) GIPAP provides assistance to patients who are not insured or reimbursed, cannot pay for treatment privately, and live in countries that have minimal reimbursement capabilities for their condition. Based on these criteria, GIPAP may cover all those diagnosed with CML in certain countries because of their low-income level. The program helps eligible patients by providing imatinib free of charge, in addition to information and referral assistance to patients, their families and caregivers through The Max Foundation.

### Data collection

Data from institutions participating in GIPAP have been collected in several databases. Axios maintains an Access to Treatment Online Management System (ATOMS) database, which captures detailed information on institutions and countries where Axios programs operate. The Max Foundation also maintains an institution, physician and a Patient Assistance Tracking System (PATS). All data analyses were retrospective, based on existing patient records collected by physicians as part of the patient’s routine clinical care and transmitted to Axios and the Max Foundation without any identifiers. Given these parameters, an Institutional Review Board (IRB) exemption was requested and received from the Western IRB. Analyses were conducted under the authorization from Novartis and the Max Foundation. In the present paper, data were available only for programs managed by Axios (44/81 countries). Consequently, programs directly managed by Novartis were not included in the analysis.

Patients’ inclusion and quarterly follow-up data were systematically collected for each hospital participating in GIPAP. All individual data were initially collected by the physicians in charge of the patients and then anonymously transmitted to Axios and the Max foundation. Axios actively monitored the quality of those individual data by conducting regular audits. At the patient level, socio-demographic and health characteristics were collected, including gender, age at enrollment, initial diagnosis (GIST or CML, along with the stage of disease: chronic, accelerated and blast crisis), and the hospital at which the patient was enrolled and treated. The date of inclusion in the cohort was the date of initial patient approval between 2003 and 2010. The end point was the date of the last reapproval in 2010 for active patients or the date of closure for other patients. The reasons for closure were: lost to contact, clinical reason, death, and other. The duration of follow up was measured from the date of inclusion to the end-point date and was considered as a proxy for survival. Only patients for whom complete institutional information was available were included in the analysis.

At the institutional level, institutions run by the government were classified as public institutions, while hospitals operated by non-governmental organizations (NGOs), or private or other organizations, were classified as private institutions. Data on core competencies related to operations research, clinical trials, and training activities were also collected. Institutions with activity in at least one of these competencies were classified as having institutional research ability. Data on institutions’ technical competencies were also collected and comprised Ph + chromosome testing for CML or CD 117 testing for GIST diagnosis, and the use of bone marrow biopsy or aspiration. Data on the availability of hematologists or oncologists at the institutions were also collected. In addition, institutions were stratified by location within the WHO region (Africa, America, Eastern Europe, Pacifica and South East Asia) in order to assess the independent role of the factors previously described, while adjusting for supranational features shared by countries from the same WHO regions; those features are related to health care system factors such as the overall financial or organizational strength, and could confound the relationship between institutional factors and enrollment or survival.

### Statistical analysis

Descriptive statistics are given as means with their standard deviation (SD), and inter-quartile range (IQR) for continuous variables and as percentages for categorical variables. In order to assess the performance of the included institutions regarding enrollment, we used linear mixed models entering the total number of enrolled patients as the dependant variable, the year as the independent variable, and the institution as a random effect so as to account for the longitudinal structure of the data based on yearly repeated measures. In order to identify institutional factors associated with higher enrollment rates, interactions were tested between the year and the following variables: public institution, presence of a hematologist or oncologist, research ability, bone aspiration capability, and location within the WHO region. The evaluation of performance at the patient level was performed using multivariate Cox models accounting for the clustered structure of the data within institutions and considering the activity status of the patient as a proxy for survival. Thus, closure for death or clinical reason was considered as the event of interest, with death determined by patient being defined as “dead” at the latest follow-up. Closure for other reasons lost to follow-up, and active status (patient still alive at latest follow-up) at the end of the study were analyzed as censored data. In an alternative sensitivity analysis, closure for any reason and lost to follow-up were also considered as events of interest. Results from the original and alternate analyses were similar, and the latter results are not included in this report. The following variables were entered as patient or institutional predictors for death: age, sex, initial phase of CML, type of institution, specialized human resources at the institution (hematologist or oncologist), research ability and the capability to perform bone marrow biopsy or aspiration, number of physicians, enrollment rate (<2, [2–5], >5 patients/year), and the WHO region. Hazard ratios (HR) were expressed with their 95% confidence intervals. Sensitivity analyses were systematically performed to assess if different results were obtained when including or excluding the programs with the highest numbers of patients (namely, Sudan [N = 971], Uzbekistan [N = 560], and Nepal [N = 540]). Because similar results were obtained, only results from the main analyses are shown. A p value < .05 was considered to be significant. Statistical analyses were performed using Stata 11.0 software package (StatCorp, TX, USA).

## Results

A total of 4,946 patients in 47 institutions within 44 countries between 2003 and 2010 were included in the study (Table 1). Of the 44 countries included, 20 were least developed countries, including 2,266 (45.8%) out of the total 4,946 patients. Institutional characteristics are summarized in Table 2. The majority of institutions (80.8%) were public/government and more than half (59.6%) were in Africa. Although most of the institutions had specialized human resources (hematologist or oncologist), institutional technical competency was low, especially with respect to the ability to perform bone marrow biopsy or aspiration. One quarter of the institutions had research capabilities.

**Table 1:** Number of patients included in GIPAP by country in 2010

**Countries**	**Patients (N = 4,946)**	**%**
Albania	84	1.7%
Armenia	167	3.4%
Azerbaijan	256	5.2%
Barbados	3	0.1%
Belarus	26	0.5%
Benin	19	0.4%
Bhutan	6	0.1%
Burkina Faso	20	0.4%
Cambodia	32	0.6%
Cameroon	53	1.1%
Côte d'Ivoire	52	1.1%
Ethiopia	279	5.6%
Fiji	15	0.3%
Gabon	9	0.2%
Georgia	252	5.1%
Ghana	69	1.4%
Haïti	8	0.2%
Kazakhstan	164	3.3%
Kenya	355	7.2%
Kyrgyzstan	105	2.1%
Madagascar	27	0.5%
Mali	48	1.0%
Mauritius	22	0.4%
Moldova	72	1.5%
Mongolia	7	0.1%
Mozambique	2	0.0%
Nepal	540	10.9%
Niger	5	0.1%
Nigeria	331	6.7%
Republic of Congo	24	0.5%
Rwanda	12	0.2%
Saint Lucia	6	0.1%
Senegal	84	1.7%
Seychelles	7	0.1%
Sierra Leone	2	0.0%
Sudan	971	19.6%
Surinam	12	0.2%
Tajikistan	1	0.0%
Tanzania	51	1.0%
Togo	38	0.8%
Uganda	90	1.8%
Uzbekistan	560	11.3%
Zambia	27	0.5%
Zimbabwe	33	0.7%

**Table 2:** Characteristics of the institutions (N = 47)

		**Institutions (N = 47)**	**%**
Public		38	80.8
WHO regions			
	Africa	28	59.6
	America	3	6.4
	Eastern Europe	9	19.1
	Pacifica	4	8.5
	South East Asia	3	6.4
Specialized human resources*			
	Hematologist and oncologist	21	44.7
	At least one hematologist or one oncologist	42	89.3
Research ability		12	25.5
Technical competency			
	Philadelphia or CD117	20	42.6
	Bone marrow biopsy or aspiration	7	14.9

*At time institutions were approved for participation in GIPAP.

Patient characteristics are summarized in Table 3. Mean patient age was 43.9 years, the sex ratio male: female was 1.3. Consistent with the predominance of African institutions, the majority of the patients were in Africa. The main diagnosis was CML in chronic phase. Average patient follow-up was 2 years and 30% of cases were closed in 5 years. Of the 30% of cases closed in 5 years, 12% of closures were due to clinical reasons, 7% due to loss of contact, 4% due to patient death and 7% due to other reasons.

**Table 3:** Characteristics of the patients (N = 4,946)

	**Patients (n = 4,946)**
Age (mean, standard deviation)	43.9 (15.7)
> = 55 years (%)	25.4
Sex ratio (male:female)	1.3
WHO regions (%)	
Africa	52.9
America	0.7
Eastern Europe	33.8
Pacifica	1.6
South East Asia	11.0
Diagnostic: CML (%)	91.4
Initial Phase (%)	
Chronic	84.7
Accelerated	13.3
Blast crisis	2.0
Status (%)	
Active	70.5
Closed	27.5
Denied	2.0
Duration of follow up (month; mean, standard deviation)	25.8 (25.9)

**Figure 1:**
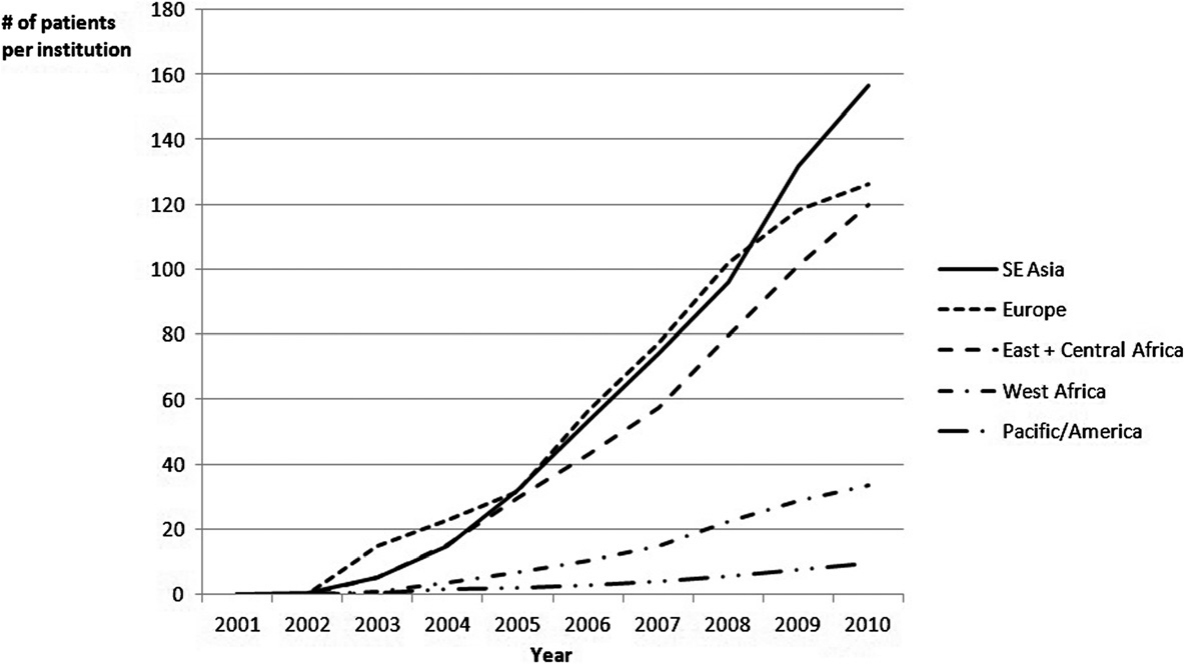
**Number of patients enrolled in GIPAP over time by WHO region: univariate analysis.**

Enrollment rates were highly variable among different institutions. The mean enrollment was 8.6 patients/year (median: 2.7; SD: 14.1; IQR: 1.2-7.4). The multivariate analysis using mixed models identified factors associated with increased enrollment (Table 4). It appeared that the WHO region was a strong predictor for the enrollment capacity (Figure 1). Eastern European and SE Asian institutions showed the highest rates of enrolling patients into GIPAP, while West African and Pacific institutions showed the lowest rates. Contrasting results were found with respect to institutional characteristics linked with expertise. Specifically, the presence of ≥1 hematologist or oncologist was associated with increased enrollment, whereas bone aspiration capability and to a lesser extent research ability were associated with lower enrollment rates. Interestingly, the number of physicians working in an individual institution was strongly associated with the number of patients enrolled when the WHO region was not considered, indicating that larger and better-staffed hospitals had the highest enrollment rates. However, this effect was no longer significant when WHO region was included in the model, mostly because Eastern Europe has predominantly larger, better-staffed hospitals compared with other WHO regions.

**Table 4:** Institutional factors associated with the number of patients enrolled per year (multivariate linear mixed model)

	**All institutions (N = 4,946 patients)**		
	**β**	**p-value**	**CI 95%**
Year	**11**.**91**	<10^-4^	[5.90; 17.93]
*Interactions*			
Year*public	2.08	0.93	[-42.92; 47.08]
Year*hematologist or oncologist	**6**.**12**	0.02	[0.98; 11.27]
Year*research	-2.59	0.13	[-5.94; 0.75]
Year*bone aspiration	-**8**.**78**	<10^-4^	[-12.89; -4.66]
Year*Europe	*0*.*00* (*ref*)		
Year*SE Asia	3.04	0.34	[-3.24; 9.33]
Year*East Africa	-0.59	0.78	[-4.86;3.67]
Year*West Africa	-**11**.**49**	<10^-4^	[-15.53; -7.45]
Year*Pacific/America	-**14**.**66**	<10^-4^	[-19.54; -9.77]

Results in **bold** are statistically significant at the 5% level.

**Figure 2:**
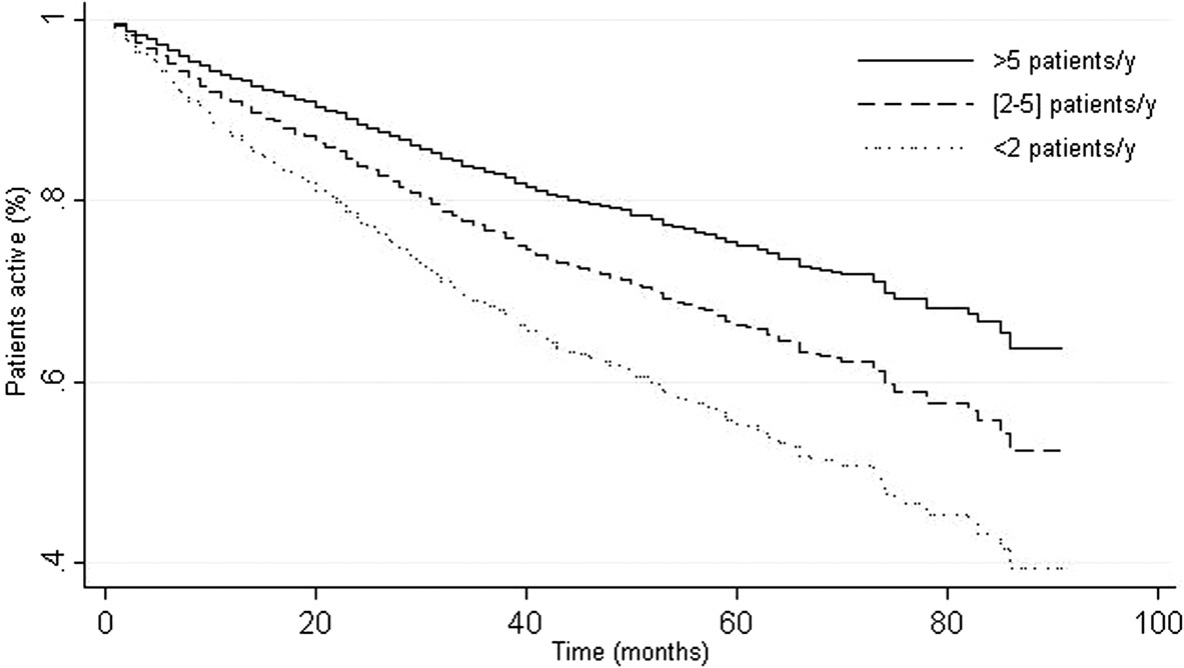
**Active CML patients as a function of institutional enrollment capacity (Cox proportional hazards regression, adjusted for age, gender, CML initial phase, public institution, presence of ≥ 1 hematologist or oncologist, research ability and technical competency).**

Table 5 shows the results from multivariate analysis assessing the patient activity as a proxy for survival. The following patient factors were identified as being associated with decreased survival: age >55 years (HR = 1.42 [1.16; 1.73]; p = 0.001) and accelerated or blast crisis at initial diagnosis (HR = 4.16 [1.87; 9.25]; p < 10^-4^). Although statistically not significant, gender was kept in the model to account for sex-related variability. At the institutional level, increased survival was found in institutions with research capabilities (HR = 0.55 [0.35; 0.86]; p = 0.01) and those with increasing enrollment ([2–5] patients/year: HR = 0.69, p = 0.09; >5: HR = 0.48, p < 10^-4^; see Figure 2). Although treatment at a public institution was associated with a trend toward decreased survival, this trend did not reach statistical significance (HR = 1.32 [0.95; 1.84]; p = 0.10). An institution’s technical competency (bone marrow biopsy or aspiration, detection of Ph + chromosome) was not significantly associated with survival. A significant interaction was found between having ≥1 hematologist or oncologist and the impact of an accelerated form of CML at initial diagnosis (HR = 0.43 [0.18; 0.99]; p = 0.05), suggesting an attenuated impact. Finally, the introduction of the WHO region into the model to control for national economics and health system characteristics shared by countries within WHO regions yielded similar results for the patient and institutional predictors (data not shown).

**Table 5:** Patient activity as a proxy for survival: role of patient and institutional factors in CML patients (multivariate Cox model)

		**Hazard ratio**	**p-value**	**CI 95%**
*Patient covariates*				
Age	<55y	*1* (*ref*)		
	> = 55y	**1**.**42**	0.001	[1.16; 1.73]
Initial phase	Chronic	*1* (*ref*)			
	Accelerated form or blast crisis	**4**.**16**	<10^-4^	[1.87; 9.25]	
*Institutional covariates*					
Research ability			**0**.**55**	0.01	[0.35; 0.86]
Public institution		1.32	0.10	[0.95; 1.84]	
≥1 hematologist or oncologist		0.88	0.73	[0.44; 1.79]	
Enrollment	<2 patients enrolled/y	*1*(*ref*)			
	[2–5] patients enrolled/y	0.69	0.09	[0.45; 1.07]	
	>5 patients enrolled/y	**0**.**48**	<10^-4^	[0.35; 0.67]	
*Interaction*					
*Accelerated form* * ≥1 *hematologist or oncologist*		**0**.**43**	0.05	[0.18; 0.99]	

Results in **bold** are statistically significant at the 5% level.

Other non-significant covariates entered in the model: gender, institution's technical competency (bone marrow biopsy or aspiration, Ph + chromosome testing), number of physicians, WHO region.

## Discussion

Institutions in Eastern Europe and SE Asia had the highest annual rates of GIPAP enrollment. The presence of ≥1 hematologist or oncologist at the time GIPAP was implemented was associated with increased enrollment. The presence of a hematologist or oncologist at an institution likely drives increased GIPAP enrollment both by drawing referrals from non-specialist physicians in the area and by providing expertise in CML and GIST that contributes to patients receiving standard of care treatment with imatinib. These factors could be used to select institutions for participation in other access programs in order to facilitate rapid enrollment of eligible patients and ensure traction of the program. Consideration of these factors will be important in deciding whether to rollout other access programs in select institutions or on a broader basis in specific countries. Similarly, these factors may help to identify the features of institutions that can best adopt CML/GIST-targeted programs and those that may need more time to become an entrenched part of the CML/GIST treatment landscape. Some factors were not assessed in our study but might play a role in explaining the variability in patient enrollment across institutions. These factors include a favorable reputation or the reference status of a center driving a high number of referrals, which are often associated with a location in the capital city of the country.

A number of patient and institutional factors were associated with decreased survival, including age >55 years, accelerated disease or blast crisis at time of diagnosis, and to a lesser extent treatment at a public hospital. Of note, treatment at an institution with high GIPAP enrollment rates was associated with improved survival. This finding could be partially explained by the increased attractiveness and higher enrollment rates found in reference centers, but might also be related to the positive impact in itself of the growing clinical experience that high-enrolling facilities gain in dealing with CML patients. The interaction between the impact of an accelerated form of CML at initial diagnosis and the availability of specialist personnel demonstrates the critical need for expertise in CML and GIST in order to optimize patient outcomes. Hematologists and oncologists are essential for ensuring that CML and GIST patients are correctly diagnosed, receive appropriate therapy for their indication, and are followed and monitored in order to detect and manage adverse side effects of imatinib therapy. Interestingly, this result was constantly found, even after entering the WHO region into the model to somewhat control for global economical and health system differences that could be nested at the supranational level and strongly influence the results. These differences include the quality and number of health care facilities, access to health care, and availability of government or private insurance programs. In order to achieve optimal health outcomes for patients in GIPAP, greater emphasis should be placed on institutional factors that improve survival, regardless of the differences in health care infrastructures among different countries.

This study had several limitations. First, a number of institutions included in this analysis had a small number of patients included, which may limit the utility of their datasets. Second, as previously described, the use of case closure as a proxy for survival is an imprecise method that may lead to incorrect assumptions about actual patient outcomes [11]. The ability to capture more precise information about the reasons for patient drop-out or case closure should improve our understanding of how various patient and institutional factors impact health outcomes for patients in GIPAP. Additionally, the study looked only at institutional factors that could affect enrollment rates. Consequently, the study does not take into account external factors, such as access to health facilities (i.e. hospitals coverage), national health policies, awareness of GIPAP, number of institutions participating in GIPAP and cultural factors, all of which could impact the number of patients seeking care for CML or GIST or the willingness of patients to participate in GIPAP. Another limitation arises from the use of raw enrollment rate as a metric for institutional success regardless of factors such as the actual number of patients the institution can reach. A small hospital would be expected to have a smaller reach and refer more patients for treatment elsewhere than a large health center that attracts more patients and receives more referrals. However, adjusting enrollment rates based on the number of physicians at an institution may account for part of the difference in reach between large and small facilities, and the stratification criterion to define high- or low-enrolling programs was only 3 patients/year (median rate among all institutions), which constitutes a low threshold reachable by most institutions, regardless of their size. Finally, our results were derived from a subset of countries and institutions within GIPAP, and did not include countries in which Novartis directly managed GIPAP programs. It is thus likely that our findings are not fully representative of all GIPAP programs, and could inadvertently highlight peculiar associations between institutional factors and the outcomes assessed. Further research should be conducted to confirm our results in other countries.

Additional data on the impact of patient and institutional characteristics on patient outcomes continue to be collected. Further evaluation of this growing body of data should help to identify factors that improve outcomes for patients with CML or GIST. Incorporation of findings of such analyses into the structure of GIPAP may help to increase the program’s effectiveness and may help improve outcomes for patients participating in the program. Identification of institutional characteristics associated with improved patient outcomes may enable criteria that could be used to select institutions for GIPAP participation. The ability to select institutions most able to improve patient outcomes would likely increase the success of an access program. This may be especially important when drug access programs are initially implemented, as early success should help them to gain traction and could facilitate their rapid and effective implementation.

The identification of infrastructure factors that contribute to the gap between the most and least successful programs may help lower-performing institutions to enroll more eligible patients into GIPAP and provide patients in the program with better quality treatment. In this study, treatment at an institution with research capabilities was associated with improved patient survival. However, such capabilities were available at only 26% of the 47 institutions evaluated. Institutional research capacity is likely a marker of scientific expertise, which is often found only in the regional referral hospital, rather than being a cause of increased survival. Patient outcomes at hospitals without research capabilities might be improved by having referral hospitals lend their scientific expertise to smaller institutions. Institutional performance may also be improved by expanding access to diagnostic tests, which could help to identify additional patients eligible for GIPAP. Similarly, better training of health care workers and strengthening of the referral system for patients diagnosed with CML or GIST may increase the opportunity for these patients to receive optimized treatment for their disease.

While GIPAP helps improve access to imatinib, the program is not designed to support these types of infrastructure improvements. Improving the skills of health care personnel and increasing the availability of diagnostic resources will require additional funding, training, and logistical support. Establishment of this infrastructure will likely require dedicated resources from and cooperation among institutions, their governments, NGOs, and manufacturers of diagnostic and research equipment. A multi-sector approach is necessary to improve access to cancer care in general and CML/GIST in particular. Support of such endeavors may provide benefit beyond the treatment of CML or GIST by improving and expanding the services available to patients with a variety of diseases.

Other programs designed to increase access to cancer medications should also consider the experiences of individual countries that have participated in GIPAP to date. Challenges implementing GIPAP have been reported, including the low priority assigned to cancer by many developing countries, the limited healthcare infrastructure and cultural and education differences [22]. In addition, the diagnostic tests required for entry into GIPAP are not available in most of the least developed countries. For example, in Kenya blood samples are sent out of the country for analysis. This puts the costs of entry tests outside the financial reach of many patients who might otherwise be eligible for the program [23]. Other challenges identified in Kenya were patients’ lack of resources to visit clinics or pickup medication and the cost of tests required to apply for second-line therapy in patients who develop resistance to imatinib [23]. These challenges are likely to occur in other low-income countries. Experiences in participating countries indicate that patient and physician education, patient monitoring and follow-up and integration with existing health care infrastructure are critical to the success of GIPAP [22].

Beyond increasing access to imatinib, the GIPAP model might also be applicable to other drugs that are indicated for CML and GIST. For example, nilotinib has been shown to provide better efficacy than imatinib in patients with newly diagnosed CML in the chronic phase [24]. Other studies have shown that nilotinib provides clinical benefit as a first-line therapy in patients with GIST [25]. Nilotinib could be an important therapeutic alternative for patients who are resistant or intolerant to imatinib and second-line sunitinib and who typically have poor prognoses and fewer treatment options [26]. Expanding access to nilotinib would further improve outcomes for CML and GIST patients in low- and middle-income countries [24,27,28].

Insights gained through further evaluation of GIPAP and the factors underpinning its success may support development of other donation and cost-sharing models that expand access to medications for the treatment of other chronic diseases in addition to cancer. Identifying sustainable methods for providing medication for chronic diseases is rising in importance given the substantial morbidity and mortality associated with cardiovascular disease, diabetes and cancer in the developing world [29]. Although GIPAP is a full donation program, the factors associated with institutional success and improved patient outcomes may help to inform the development of other, more economically sustainable models for increasing access to medicines for cancer and other chronic diseases.

## Conclusion

In the context of GIPAP, treatment at an institution with high enrollment rates, local medical expertise and research capabilities are associated with improved patient survival, regardless of the patient’s age or initial stage of the disease. Use of these findings to optimize GIPAP should help improve access to care and treatment outcomes for patients with CML or GIST.

## Competing interests

This work was funded via an unrestricted grant from Novartis to the Axios Foundation. This applies to JS. Other authors have declared no conflicts of interest.

## Authors’ contributions

All authors participated in the study conception and design. ET and PGG participated in the data acquisition and extraction. EA, MPT and JL performed the statistical analysis and interpretation of data. ET, JS and EA made the drafting. All authors participated in the critical revision of the manuscript and have read and approved the final manuscript.

## Pre-publication history

The pre-publication history for this paper can be accessed here:

http://www.biomedcentral.com/1472-6963/13/304/prepub
